# Exercise and Nutrition Interventions in Patients with Head and Neck Cancer during Curative Treatment: A Systematic Review and Meta-Analysis

**DOI:** 10.3390/nu12113233

**Published:** 2020-10-22

**Authors:** Asta Bye, Jon A. Sandmael, Guro B. Stene, Lene Thorsen, Trude R. Balstad, Tora S. Solheim, Are Hugo Pripp, Line M. Oldervoll

**Affiliations:** 1 Faculty of Health Sciences, OsloMet—Oslo Metropolitan University, 0130 Oslo, Norway; apripp@oslomet.no; 2 Regional Advisory Unit for Palliative Care, Department of Oncology, Oslo University Hospital, 0424 Oslo, Norway; 3 Unicare Røros, Øverhagaen 15, 7374 Røros, Norway; jon.arne.sandmael@unicare.no; 4 Department of Cancer Research and Molecular Medicine, Faculty of Medicine and Health Sciences, Norwegian University of Science and Technology (NTNU), 7491 Trondheim, Norway; guro.b.stene@ntnu.no (G.B.S.); trude.r.balstad@ntnu.no (T.R.B.); tora.s.solheim@ntnu.no (T.S.S.); 5 Cancer Clinic, St. Olavs Hospital, Trondheim University Hospital, 7006 Trondheim, Norway; 6 National Advisory Unit on Late Effects after Cancer Treatment, Department of Oncology, Oslo University Hospital, 0424 Oslo, Norway; LKA@ous-hf.no; 7 Department for Clinical Service, Division of Cancer Medicine, Oslo University Hospital, 0424 Oslo, Norway; 8 Oslo Centre of Biostatistics and Epidemiology, Research Support Services, Oslo University Hospital, 0424 Oslo, Norway; 9 Center for Crisis Psychology, Faculty of Psychology, University of Bergen, 5020 Bergen, Norway; line.oldervoll@ntnu.no; 10 Department of Public Health and Nursing, Faculty of Medicine and Health Sciences, The Norwegian University of Science and Technology (NTNU), 7491 Trondheim, Norway

**Keywords:** head and neck neoplasms, nutrition, physical exercise, radiotherapy, meta-analysis, nutritional status, physical function, body composition

## Abstract

The aim of this meta-analysis was to examine the effects of nutritional and physical exercise interventions and interventions combining these interventions during radiotherapy treatment for patients with head and neck cancer on body composition, objectively measured physical function and nutritional status. Systematic electronic searches were conducted in MEDLINE (PubMed interface), EMBASE (Ovid interface), CINAHL (EBSCO interface) and Cochrane Library (Wiley interface). We identified 13 randomized controlled trials (RCTs) that included 858 patients. For body composition, using only nutrition as intervention, a significant difference between treatment and control group were observed (SMD 0.42 (95CI 0.23–0.62), *p* < 0.001). Only pilot RCTs investigated combination treatment and no significant difference between the treatment and control groups were found (SMD 0.21 (95CI −0.16–0.58), *p* = 0.259). For physical function, a significant difference between treatment and control group with a better outcome for the treatment group were observed (SMD 0.78 (95CI 0.51–1.04), *p* < 0.001). No effects on nutritional status were found. This meta-analysis found significantly positive effects of nutrition and physical exercise interventions alone in favor of the treatment groups. No effects in studies with combined interventions were observed. Future full-scaled RCTs combining nutrition and physical exercise is warranted.

## 1. Introduction

Head and neck cancers (HNCs) comprises malignancies of the oral cavity, throat, larynx, salivary glands as well as nasal and paranasal sinuses. Surgery and radiotherapy (RT), sometimes combined with chemotherapy (CT) are the main treatment approaches [1]. Aggressive treatment regimens are effective to achieve tumor control and cure patients, but they also cause severe side-effects such as mouth dryness, mucositis and difficulties in swallowing [2]. Eating challenges due to tumor growth is one of the presenting symptoms of HNCs for many patients. Not surprisingly, when the challenges of the tumor is amplified by side effects of treatment that compromise dietary intake, many patients experience unintentional weight loss accompanied with muscle wasting [3]. Muscle wasting may influence muscle function and lead to loss of strength, increase fatigue and decrease quality of life [4].

To counteract the negative effects of weight loss and diminishing muscle mass for patients with HNCs during RT, it is recommended to ensure nutritional intake primarily through nutritional counseling and/or use of oral nutritional supplements (ONS) [5,6]. These recommendations are based on reviews indicating that dietary counselling can improve nutritional status and quality of life during RT [7,8]. However, the evidence supporting these strategies are inconclusive partly because previous reviews were not limited to randomized controlled trials (RCTs), patients with HNCs or interventions starting simultaneously with anticancer therapy. Physical exercise is another strategy that has the potential to decrease muscle catabolism and increase anabolism [5]. For patients with HNCs, exercise interventions have been tested in several pilot studies and are shown to be feasible, safe and to have potential impact on body composition, physical function, quality of life and fatigue management [9,10].

For patients treated for HNCs, weight loss, loss of strength, fatigue, and decreased quality of life are parts of a multidimensional problem related to both inadequate food intake and inactivity [7,11,12]. It is therefore a need to examine the impact of interventions combining nutrition and physical exercise as well as the feasibility of such interventions in this exposed population. Most previous studies have focused on either nutrition or physical exercise. However, physical exercise may be of importance for full effect of nutritional interventions and vice versa, sufficient nutrition is essential for optimal effect of physical exercise [5]. It could thus be hypothesized that a treatment approach including both nutrition and exercise is more effective improving patient outcomes than each intervention given alone. The aim of this systematic review and meta-analysis is therefore to examine current evidence for nutritional interventions alone, physical exercise interventions alone and interventions combining nutrition and physical exercise during radiotherapy treatment for patients with head and neck cancer. The main research questions are: (1) What are the effects on nutritional status, body composition and objectively measured physical function? (2) What is the content of the interventions? (3) What is adherence to and completion rate of the different interventions?

## 2. Material and Methods

The present review was conducted in accordance with the Preferred Reporting Items for Systematic Reviews and Meta Analyses (PRISMA) statement guidelines [13] and the review protocol is registered in the International Prospective Register of Systematic Reviews (PROSPERO) (Reg. nr.; CRD42018081487).

### 2.1. Data Sources, Search Strategy and Selection Criteria

#### 2.1.1. Data Sources

Electronic searches were conducted the 31 October 2018 in MEDLINE (PubMed interface), EMBASE (Ovid interface), CINAHL (EBSCO interface) and Cochrane Library (Wiley interface). Additionally, the reference list of included studies and relevant systematic reviews were screened. Updated search in MEDLINE for the period between the 1 November 2018 and 3 June 2019 was later conducted to identify any additional relevant publication.

#### 2.1.2. Search Strategy

The searches consisted of combinations of controlled terminology and free-text terms expressing the concepts (1) head and neck cancer and (2) exercise and (3) nutrition, adapted to each specific database. In Appendix A, the full search strategy of each database is described.

#### 2.1.3. Selection Criteria

Inclusion criteria are shown in Table 1. Full scale RCTs and pilot RCTs evaluating the feasibility and/or effect of nutritional interventions and/or physical exercise published in peer-reviewed journals were considered for inclusion.

**Figure 1:**
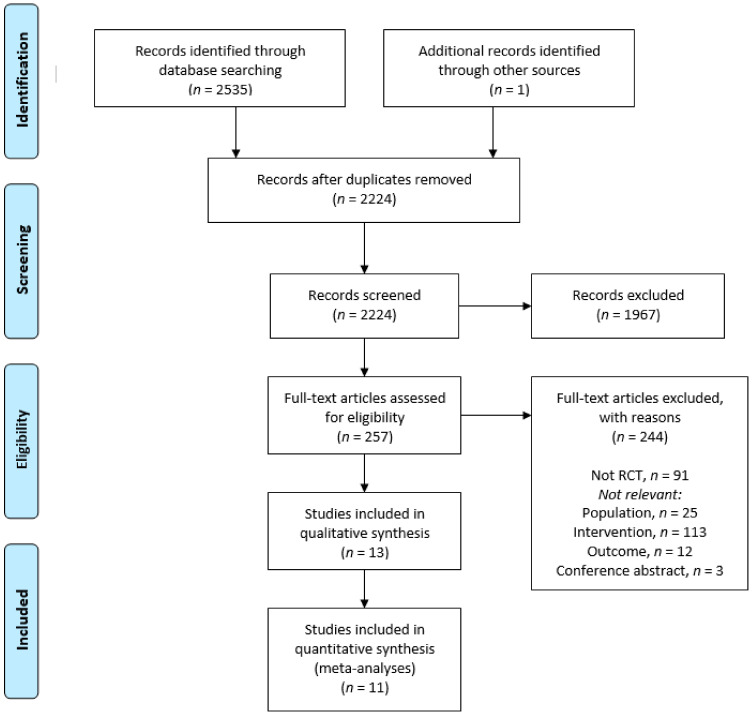
Preferred reporting items for systematic reviews and meta-analyses (PRISMA) flow diagram of reviewed and included studies.

The details of the search process are shown in Figure 1. All identified records were screened for duplicates and irrelevant titles by the second author (JAS). Remaining abstracts were screened by three pairs of reviewers (LMO/LT, AB/TSS and GBS/JAS) and full-text papers were subsequently screened by the same pairs. Reasons for excluding abstracts and full-text papers were documented by the pairs. A third reviewer’s opinion was called for in cases of disagreement regarding eligibility. Data concerning participant characteristics, content of the interventions, outcome measures, results and conclusions were extracted. Disagreement on final inclusion and exclusion were agreed by consensus by three of the authors (JAS, LMO, AB).

### 2.2. Quality Assessment

The methodological quality of the included RCTs was assessed independently by two of the reviewers (AB, LMO), using the Physiotherapy Evidence Database (PEDro) scale [14]. The PEDro scale is developed for the Physiotherapy Evidence Database by the Centre for Evidence-Based Physiotherapy to evaluate the methodological quality of studies with physical exercise and therefore relevant for this review. The scale is also found to have acceptable reliability and validity when examining studies with other types of interventions [15]. The PEDro scale examines presence or absence of 11 quality measures, but only 10 are scored leaving the final score ranging from 0 to 10 points [14]. Criterion one relates to external validity (not calculated), criteria 2–9 assess internal validity, and criteria 10 and 11 verify whether the studies have enough statistical information for the results to be interpretable. A score between 8–10 is considered as high quality, 5–7 as moderate quality and 0–4 low quality.

### 2.3. Data Extraction and Statistical Analyses

The following data was extracted from the included studies by the first author (AB): authors, year, country, study design, patient group (sample size and disease), inclusion criteria, details of the interventions, adherence to the intervention, completion rate, outcomes and results. Adherence was defined to be reflected by measures of how well the patients complied with the intervention, e.g., energy intake in relation to calculated needs and number of exercise sessions completed. Whether the patients stayed in the trial to the end of study was registered as completion. Outcomes of interest were nutritional status, body composition and objectively measured physical function. Regarding nutritional status were use of validated assessment instruments (generic and disease specific) considered relevant as well as use of medical data known to reflect nutritional status. Regarding body composition, the following measures were considered relevant; absolute or change in body weight, body mass index (BMI), muscle mass, lean body mass or fat mass. For objectively measured physical function, the following measures were relevant; absolute or change in any physical fitness test such as a walk test, handgrip strength, physical or performance battery. When data were available and reliable scales were used, the studies were combined in a meta-analysis. We attempted to contact study authors to request values for any missing data and if this was not successful, we did not impute the data into the meta-analysis.

Publication bias was assessed by funnel plots and the *LFK* index and *Doi* plots [16] to detect and quantify asymmetry of study effects. LFK index values outside the interval between −1 and +1 are considered consistent with asymmetry (i.e., publication bias). Stata version 15 (Stata Corp, College Station, TX, USA) with the user-developed packages metan [17], metafunnel [18] metabias [19] and Doi plot and estimates the LFK index [20] were used for all the estimations.

## 3. Results

Search results are summarized in the Preferred Reporting Items for Systematic Reviews and Meta-Analyses (PRISMA) flow diagram (Figure 1). The database searches retrieved a total of 2535 records. One additional record was identified from a hand search of review paper references. After removal of duplicates, 2224 studies were left to screen. After screening title and abstracts, 1967 papers were excluded for not meeting the inclusion criteria, leaving 257 studies for full text review. After the full text review, 13 RCTs were included (Table 2), nine full scale RCTs and four pilot RCTs. Reasons for exclusions are listed in Figure 1. The included studies were conducted in Europe (*n* = 4), United States of America (*n* = 4), Asia (*n* = 3), Canada (*n* = 1), and Australia (*n* = 1). Publication year from 1984 [21] to 2019 [22].

### 3.1. Quality Assessment

Two of the full scale RCTs studies [23,26] were considered as high quality (eight to 10 points) (Table 3), four were considered of moderate quality (five to seven points) [22,27,29,30] and three as low quality [21,24,25]. Two of the pilot RCTs [28,31] were of high quality and two of moderate quality [9,32]. All the high and moderate quality studies clearly specified methods used and how the randomization was performed. Methodological uncertainties included blinding (minding that it is difficult to blind participants in both nutritional and exercise studies) and lack of intention to treat analysis [23,26,28]. For the three low quality studies, eligibility criteria were not specified [24] or unclear [25], it was uncertainties about random allocation to groups and similarity in outcome variables at baseline [21,24] as well as uncertainties regarding concealed allocation and intention-to-treat analysis [21,24,25].

### 3.2. Study Characteristics

Four pilot RCTs (Table 2) investigated effects of interventions combining nutrition and physical exercise [9,28,31,32] with sample sizes between 15 [28] and 60 participants [32]. Seven studies investigated the effects of nutritional interventions only [21,23,24,25,26,27,29] with sample sizes varying from 31 [24] to 159 participants [23] and study duration ranging from six weeks during treatment [26] up to six months due to follow up after the intervention period [21] (Table 2 and Table 4). One study had three arms [27], i.e., one group received individualized dietary counselling, one ONS and the last group was advised to eat ad libitum. Two studies with sample sizes of 48 and 148 patients investigated exercise interventions during RT with follow up seven and four weeks after end of RT, respectively [22,30] (Table 4).

### 3.3. Effects on Nutritional Status, Body Composition and Physical Function

Outcomes and effects of the interventions are summarized in the Supplementary table. Nutritional status was measured in three studies [26,27,32]. No statistically significant difference between intervention and control group were found. Just two of the studies [26,32] presented group data and therefor a quantitative analysis of effects on nutritional status was not meaningful.

**Figure 2:**
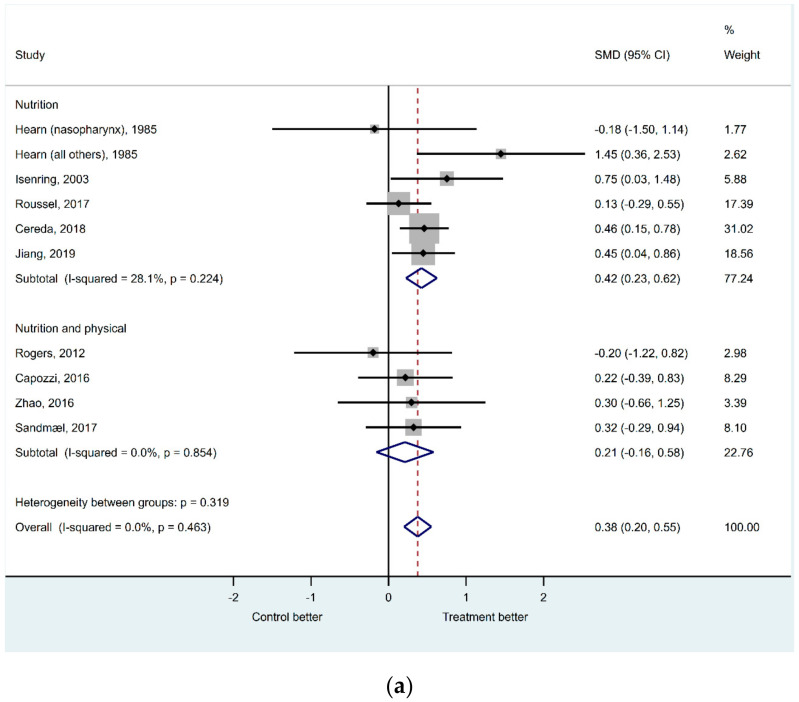

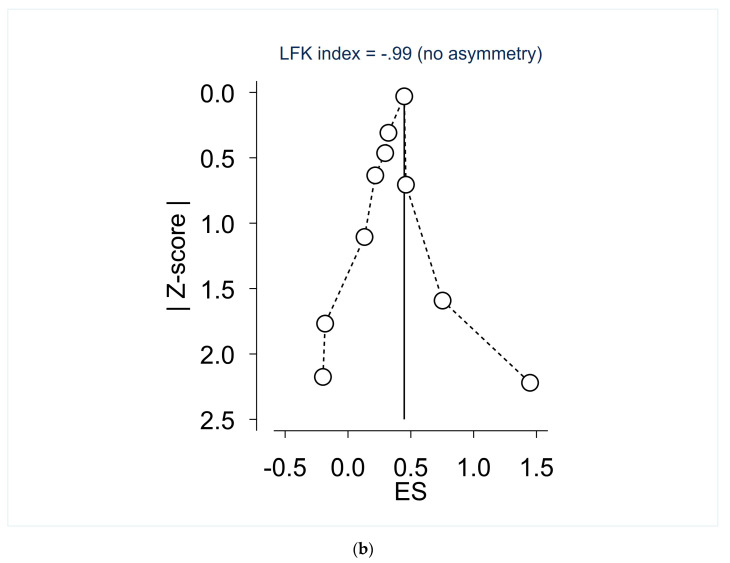
(**a**) The effects of nutritional and exercise interventions on body composition; (**b**) the symmetry of body composition results presented in Doi plot. ES, effect size.

Nine studies were included in the quantitative synthesis of effects on body composition (Figure 2a) [9,23,24,25,26,28,29,31,32]. Absolute weight or weight change were used as outcome variable for body composition in all studies except for three [28,31,32] were change in BMI was used. In the fixed-effect meta-analysis on body composition, it was a significant difference between intervention and control group for the studies using only nutrition as intervention (SMD 0.42 (95CI 0.23 – 0.62), *p* < 0.001), but not for the trials combining nutrition and physical exercise (SMD 0.21 (95CI −0.16 – 0.58), *p* = 0.259). Still, the estimated difference using all the included trials was highly significant (SMD 0.38 (95CI 0.20 – 0.55), *p* < 0.001) with a better outcome for the intervention group. The heterogeneity was low with an overall I^2^ statistics of 0% and a non-significant Cochran’s Q test (*p*-value = 0.463). Assessing the corresponding funnel plot (Supplementary Figure S1) and the Egger’s test for small-study effects (*p* = 0.947) as well as the DOI plot and the LFK index (−0.99) (Figure 2b), no publication bias in the studies were detect.

**Figure 3:**
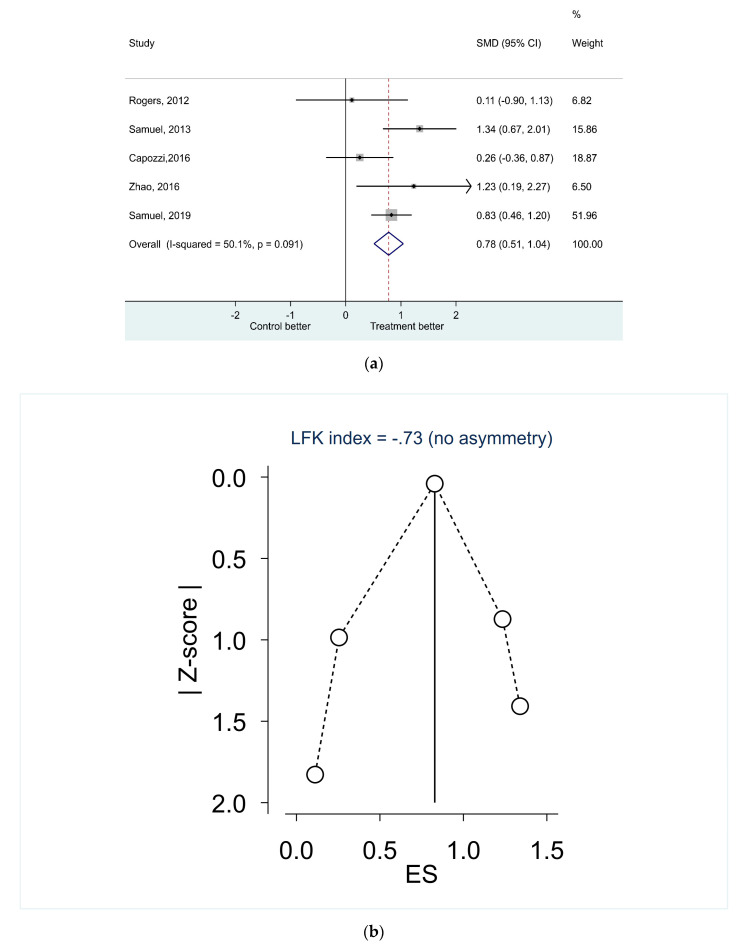
(**a**)Effects of nutritional and exercise interventions on physical function; (**b**) the symmetry of physical function results presented in Doi plot.

Five studies were included in the quantitative synthesis of effects on physical function (Figure 3a) [22,28,30,31,32], of which three studies combined nutrition and physical exercise [28,31,32]. The six minutes-walk test was used as outcome measure except for Capozzi et al. [32] and Rogers et al. [28] where handgrip strength was used. In the fixed-effect meta-analysis on physical function, it was a highly significant difference between intervention and control group (SMD 0.78 (95CI 0.51–1.04), *p* < 0.001) with a better outcome for the intervention group. The heterogeneity was higher than in the trials on body mass with an overall I^2^ statistics of 50.1%, but a non-significant Cochran’s Q test (*p*-value = 0.091). Assessing the corresponding funnel plot (Supplementary Figure S2) and the Egger’s test for small-study effects (*p* = 0.896) as well as the DOI plot and the LFK index (−0.73) (Figure 3b), no publication bias in the studies were detected.

### 3.4. The Content of the Interventions

A detailed description of the content in the interventions is presented in Table 4.

#### 3.4.1. Nutrition

The most frequent nutritional intervention (six of 11 studies) was individualized dietary counselling based on regular food with or without ONS aiming to meet estimated individual needs for energy and protein [23,25,27,28,29,31]. In three of the studies [23,25,29], dietary counselling was considered as standard care and therefor applied in the control group but with less monitoring and feedback than in the intervention group. In one study, participants in both groups received dietary counselling by a dietitian before initiation of CRT, and then again at discretion of the attending physician, if the participants experienced a decrease in BMI of 5 to 10% [31]. Two studies intervened with ONS only and the patients were encouraged to take 1-2 bottles each day [9,26]. Nasogastric tube feeding was the intervention in two studies whereas the control group received dietary counselling [21,24]. For both tube feeding and counselling, the goal was to meet estimated energy (40 kcal/kg/day) and protein (1 to 1.5 g protein/kg/day) needs. For the last study [32] the exact content of the nutritional intervention was not specified, but it was reported that a group based dietary counselling was given by a dietitian as part of a 12-week lifestyle program.

#### 3.4.2. Physical Exercise

In four of the six studies that included physical exercise, a combination of resistance and aerobic exercises was applied [9,22,30,31]. Two studies intervened with resistance exercises only [28,32]. The resistance exercises covered the major muscle groups and were monitored and supervised by a trainer or physiotherapist 2–5 times a week during RT in five studies [9,22,28,31,32]. In the last study [30] the patients received an individualized and structured exercise program, and their family members were asked to motivate the patients to do the exercises. In all studies, the patients were encouraged to proceed with the resistance exercises at home after RT and a weekly follow up telephone was applied in three studies [22,28,31]. The applied aerobic exercise included brisk walking for 15–20 min five days a week [22,30], multiple short duration continuous walking to achieve a total walking time of 30 min a day [31] and 150 min moderate intensity aerobic exercise per week [9].

### 3.5. Adherence to the Intervention and Completion Rate

Adherence to the interventions and completion rates are presented in Table 4.

#### 3.5.1. Adherence

Four studies [25,30,31,32] did not present data regarding nutritional interventions adherence. Three studies evaluated adherence in relation to how well the patients met their energy and protein needs [23,27,29]. In two of these studies [23,29] intake in accordance with estimated needs was reported during the intervention and follow-up both in the intervention and control group. Ravasco et al. [27] found that the group receiving dietary counselling had higher energy intake and thereby better adherence than the group using ONS and the ad lib group at the end of RT and at three months follow-up. In the two studies using ONS, adherence was evaluated as number of ingested ONS in relation to planned amount. One study reported an adherence rate of 57% during treatment and 76% after treatment [9] while the other study reported that about 52% of the provided ONS were consumed [26]. In the two studies investigating tube feeding, 9% [24] and 14% [21] refused the intervention and were converted to the control group. In the same studies, were two patients converted from the control to the intervention group in each study due to weight loss during the first week of RT.

Four of six studies reported data on adherence to the exercise intervention [9,28,31,32]. Rogers et al. [28] reported that 83% of the planned exercise sessions were completed at 6 weeks and 62% in the period between week 6 and 12. In Zhao et al., [31] the overall adherence to the exercise program was 72% (15.2 out of maximum 21 sessions). Two studies had a similar design with an intervention during RT and another group receiving a delayed intervention, i.e., after completion of RT [9,32]. In the study of Capozzi et al. [32] the weekly attendance to the supervised exercise program was 45% during cancer treatment and 61% after. In Sandmael et al. [9] overall adherence to strength and aerobic exercise was 81% and 94%, respectively.

#### 3.5.2. Completion Rates

All studies reported data on completion rate. Ravasco et al. [27] reported that all patients completed the study. For the other studies with nutritional intervention the completion rate varied between 70% [23] and 92% [21]. For most nutritional studies patients lost for follow up was similar in the intervention and control group except for the study of Isenring et al. [25] (7% in the intervention and 14% in the control group) and Hearne et al. [24] (22% in the intervention and 8% in the control group).

The exercise only studies reported 81% [22] and 86% [30] completion and similar number of patients lost for follow-up in the intervention and control group. In the feasibility studies investigating a combination of nutrition and exercise, the completion rate varied between 60% [32] and 87% [28].

## 4. Discussion

This systematic review and meta-analyses show that nutrition and physical exercise interventions have a positive effect on body composition and physical function for patients with HNCs undergoing RT (+/- concomitant CT) with a curative intent. The nutritional interventions were mainly individualized dietary counselling aiming to meet estimated energy and protein needs and use of ONS in case of inadequate energy intake. The physical exercise was typically supervised with a combination of strength and aerobic exercises used, performed two to five times a week. In case of nutritional interventions, the adherence to dietary advices after counseling was reported good, but it was measured in just half of the studies. When ONS were used, about half of the patients did not consume the recommended amount. The adherence to exercise varied between 45% and 83% and completion rates between 60% and 80%. The lowest adherence and completion rate were reported for interventions combining nutrition and physical exercise.

### 4.1. Strengths and Limitations

This is, to our knowledge, the first systematic review seeking to examine the effects of both nutrition and physical exercise in patients with HNCs undergoing RT. A major strength of this review is the authors’ attempt to identify all relevant studies by using a comprehensive search strategy in multiple databases lead by a research Liberian as well as methodological strictness performing the systematic review and meta-analyses. All authors participated in the process which also included hand-search of review paper references to identify additional studies that may have been lost in the initial search.

Based on available guidelines, it was expected that interventions combining nutrition and physical exercise would have a better effect on nutritional status, body composition and physical function than nutrition and physical exercise alone [5]. However, only four studies [9,28,31,32] with combined interventions were identified and included in the meta-analysis to explore the effects on body composition. A major limitation is that all four studies were pilot/feasibility, i.e., not powered to detect statistically significance difference between the groups. Another limitation was that no relevant measures regarding the other outcomes of interest, nutritional status and physical function, were provided. Based on this, it is not possible to draw any meaningful conclusion regarding effects of combined nutrition and physical exercise interventions in patients with HNCs undergoing RT.

Several factors, largely reflecting limitations in the included studies, may have influenced the results showing effect on body composition and physical function of the interventions. Nine of 13 studies were of poor or modest methodological quality mainly due to uncertainties about baseline assessments of outcome variables, heterogeneity in anti-cancer treatment and random allocation to groups. In addition, uncertainties regarding intention-to-treat analysis were seen in six of the studies. The lowest methodological quality was seen in the oldest studies [21,24,25,27], all investigating effects of nutritional interventions. One of these studies used only within group and not between-group statistical comparisons analyzing the outcomes of interest for this review [27] which make the results more or less useless in a randomized design where the aim is to compare two or more groups.

The specific interventions given in the included studies were heterogenous and in many studies poorly described. Even if individualized dietary counselling and combinations of strength and aerobic exercises were the most common interventions, it was a variation in the delivery that may have affected the results. In one of the studies [31] the nutrition intervention was delivered as part of a comprehensive lifestyle program. Thus, participants were receiving concomitant additional lifestyle interventions such as clinical support and health education which may have had a synergistic effect on the outcomes. Additionally, parts of the lifestyle program were also used in the control group potentially contributing to an equalization of possible effects [33].

The measurements of outcomes of interest for this review were highly heterogeneous. In the nutrition field it is an acknowledged problem that high quality indicators to demonstrate the effect of nutritional interventions are lacking [34]. Changes in weight and BMI have long been regarded as practical indicators of changes in nutritional status and body composition [35]. Although of value, these measurements do not captured changes in muscle mass which is associated with several negative outcomes specifically in cancer patients [35,36]. The use of weight and BMI as measures of body composition may have confounded the effects of the nutrition intervention but may even more the interventions with physical exercise since they are expected to have a direct effect on muscle mass. The exercise studies were also heterogenous regarding the measurements of physical function (three used six-minute walk test and two used hand grip test) and one study [9] did not include an objective physical functioning at all. The most used six-minute walk test mainly measure walking ability and endurance and may not catch up changes in muscular strength, muscle mass and muscle waste [37,38]. Thus, future full scaled studies including both nutrition and physical exercise are warranted. The future studies should more carefully choose an appropriate and specific method to measure body composition and physical function according to the intervention given.

### 4.2. Nutritional Interventions

The result showing that nutritional interventions alone have a positive effect on body composition is in line with the results from a former study reviewing effects of nutritional interventions on nutritional status, quality of life and mortality in patients with HNCs receiving RT [7]. The authors concluded that individualized dietary counselling based on regular food with or without ONS has a beneficial effect on energy and protein intake and nutritional status when comparing with standard nutritional care. Additionally, they found that ONS alone only showed short-term effects on energy and protein intake and inconsistent effects on nutritional status and tube feeding versus ONS showed no beneficial effects.

The current nutrition guidelines for patients with HNCs recommends individualized dietary counselling in combination with ONS and/or initiation of tube feeding when oral intake is inadequate [5]. In the present review, this approach was used in five of 11 studies with nutritional intervention [23,25,26,29,31] while one used only dietary counselling [28] and a pilot study used only ONS [9]. Dietary counselling is considered the best approach to promote adherence to dietary advices [39,40] since it allows an individual tailoring of the diet to personal needs and desires [40,41]. An indication of this was also found in one of the selected studies, designed to compare effect on dietary intake after dietary counselling, use of ONS and eating ad libitum [27]. It was concluded that dietary counselling was the only intervention that improved dietary intake and had a positive effect on nutritional status. However, a more recent study found that HNCs patients receiving counselling in combination with ONS had a higher intake of micronutrients and preserved weight better than patients not using ONS [42], supporting the current guidelines recommending addition of ONS when oral intake is inadequate [5].

Two older studies of low methodological quality used tube feeding from start of RT [21,24]. A recent review did not show that prophylactic tube feeding in patients with HNCs is more beneficial than ONS regarding nutritional status and body composition [7]. However, after individual considerations tube feeding may be regarded beneficial, but since some patients may consider it burdensome, it is important to explore the patient’s wishes and preferences before initiated [43,44].

Unfortunately, there was little information about adherence to dietary counselling, as it was reported in only three of six studies [23,27,29]. All studies reported dietary intake in accordance with estimated needs indicating high adherence. This supports the assumption that individualized dietary counselling promote adherence to dietary advices [40]. The study of Ravasco et al. [27] also found that counselling resulted in a higher intake of macronutrients than just using ONS. This is in line with the findings from the two included studies using ONS as intervention [9,26], both showing low adherence (57% and 52%, respectively). In a qualitative study from our group the respondents with HNCs expressed that ONS only made sense during the initial weeks of radiotherapy, and that after this it got unbearable to ingest them due to side effects from RT [45]. These respondents also indicated that being exposed to the side effects of radiotherapy was experienced as quite different from just hearing and reading about them. This finding may have consequences for when nutritional interventions should be delivered. It is possibly not necessary to use intensive dietary counselling from start of RT, but instead use nutritional surveillance systematically and provide of dietary counselling when the patients developed eating problems as recommended in a study [46].

### 4.3. Exercise Interventions

According the recently published guidelines for physical exercise in cancer patients, there is relative strong evidence for prescribing physical exercise for the effects on physical function for cancer patients [47]. However, it should be noted that these guidelines are based on data from self-reported physical function (using different self-reported questionnaires) and not results for objective physical function being used as the outcome in this meta-analysis. Regarding data from objective measures, the evidence base on this outcome remains immature and more challenging to aggregate due to the variation and limitations of assessment techniques. Therefore, the results from our meta-analysis needs to be regarded with caution and more studies are warranted to conclude more firmly.

Four of six potential studies reported adherence to the exercise intervention, and reported adherence was in general high, but ranging from 45–83%. However, the reporting of exercise was different between the studies, making it challenging to compare. Sandmæl et al. [9] reported a high adherence rate of 81% for the entire period during treatment, while Rogers et al. [28], divided the adherence rate in the period between 0–6 weeks (83%) and 6–12 weeks (62%) showing a decline in adherence in the six last weeks of treatment as the patients get more complaints. The reporting of adherence in exercise studies has until recently been suboptimal in most studies and in the future, greater demands should be made concerning reporting of adherence [37].

Supervised exercise appears to be more effective than unsupervised or home-based interventions [47,48]. In line with these recommendations, all the included studies in this meta-analysis used supervised exercise. 

## 5. Conclusions

This meta-analysis found significantly positive effects of interventions with nutrition alone and physical exercise alone in body composition and objective physical function in favor of the treatment groups. However, the included studies were highly heterogenic both regarding measurement methods and the content of the interventions which may have affected the result of the meta-analysis. Due to the pilot and feasibility design of the studies combining physical exercise and nutrition, no conclusions can be drawn concerning the effects from these studies. Future full-scaled RCTs combining nutrition and physical exercise is warranted.

## Figures and Tables

**Table 1:** PICOS (patients/population, intervention, comparator, outcomes, study design) criteria for inclusion and exclusion of studies.

	Inclusion Criteria	Exclusion Criteria
**Population**	Adults diagnosed with HNC, receiving RT with curative intent (± concomitant CT)	Patients <18 years of age, cancer with another origin, surgery as only treatment
**Intervention**	(1) Physical exercise or (2) nutrition or (3) a combination of exercise and nutrition. Initiated at start of RT and conducted during RT. Physical exercise is defined as sessions of muscle strength and/or aerobic exercise. Nutrition is defined as, dietary counselling, oral nutritional supplements or enteral nutrition by nasogastric tube or PEG	(1) Interventions initiated before start or after completion of RT (2) Nutritional interventions consisting only of vitamins or minerals (3) Comparisons of enteral and parenteral solutions (4) Swallowing exercise interventions alone
**Comparator**	Standard care or placebo	
**Outcome**	Nutritional status (validated assessment instruments, e.g., SGA or PG-SGA), body composition (body weight, BMI, muscle mass or lean body mass, fat mass) and/or objectively measured physical functioning (walk test, handgrip strength, physical or performance battery)	Quality of life, fatigue, feasibility, treatment tolerance or survival as only outcome measure
**Study design**	RCTs or pilot RCTs	Case series with <10 participants, qualitative studies, reviews, letters, editorials, notes
**Setting**	No restrictions	
**Time frame**	No restrictions	

Abbreviations: HNC: head and neck cancer; RT: radiotherapy; CT: chemotherapy; PEG: percutanous endoscopic gastrostomy; SGA: subjective global assessment; PG-SGA: patient generated-SGA; BMI: body mass index RCT: randomized clinical trial.

**Table 2:** Characteristics of the included studies organized alphabetically.

Author, Year	Country	Study Design	Type of Intervention	Sample Size	Age in Years	Clinical Info Included Patients	Cancer Treatment
Capozzi, 2016 [10]	Canada	Pilot RCT	Exercise and nutrition	60 male 82%	Mean (SD) 56.1 (9.2)	Diagnosis (n): Larynx-hypopharynx (6), Nasopharynx (4), Oral (8), Oropharynx (29), Other (6), Unknown origin (7) Stage (n): I-III (11), IV (48) Histology: NA	RT (*n* = 16) CRT (*n* = 44)
Cereda, 2018 [23]	Italy	RCT	Nutrition	159 male 72%	Mean (SD) Counselling 63.8 (12.7); counselling + ONS 66.5 (14.5)	Diagnosis (n): Hypopharynx (13), Larynx (41), Naso- oropharynx (44), Oral (29), Other (32) Stage (n): 0-II (76), III (40), IV (34) Histology (n): Squamous (124), Lymphoma (20), other (15)	RT (*n* = 98) and CRT (*n* = 61)
Daly, 1984 [21]	USA	RCT	Nutrition	40 male 80%	Mean (SD), Intervention 53 (15) Control 55 (13)	Diagnosis (n): Nasopharynx (15), Other (25) Stage (n): II (2), III (9), IV (28) Histology: NA	All RT
Hearne, 1989 [24]	USA	RCT	Nutrition	31 male 77%	Mean (range), Intervention 52.1 (22–74) Control 56.1 (37–83)	Diagnosis (n): Nasopharynx (9), Other (22) Stage (n): III (11), IV (20) Histology: NA	All RT
Isenring, 2003 [25]	Australia	RCT	Nutrition	36 male 81%	Mean (SD), 63 (15)	Diagnosis: NA Stage: NA Histology: NA	All RT
Jiang, 2018 [26]	China	RCT	Nutrition	100 male 69%	Mean (SD), Intervention 46.7 (10.9)) Control 48.2 (11.1)	Diagnosis: Nasopharynx Stage: III (43), IVa-b (57) Histology: NA	All CRT
Ravasco, 2005 [27]	Portugal	RCT	Nutrition	75 male 80%	Mean (SD) 60 (11)	Diagnosis (n NA): Larynx, Oropharynx, Nasopharynx, Tongue Stage (n): I-II (30), III-IV (45) Histology: NA	All RT (all pre-vious CT)
Rogers, 2013 [28]	USA	Pilot RCT	Exercise and nutrition	15 male 80%	Mean (SD) 60.5 (12.5)	Diagnosis (n): Nasopharynx, scalp and salivary glands (5), Other (10)Stage (n): I-II (7), III -IV (8) Histology: NA	RT (*n* = 11), CRT (*n* = 4)
Roussel, 2017 [29]	France	RCT	Nutrition	87 male 81%	Mean (SD) 60 (10)	Diagnosis (n): Hypopharynx (11), Larynx (19), Oral (9), Oropharynx (40), Nasopharynx (2), Sinus (2), Unknown origin (4) Stage (n): I-II (14), III (14), IV-V (59) Histology: NA	RT (*n* = 28), CRT (*n* = 59)
Sandmaell, 2017 [9]	Norway	Pilot RCT	Exercise and nutrition	41 male 61%	Mean (SD) 63.2 (9.3)	Diagnosis (n): Larynx (4), Nasal (1), Oral (5), Pharynx (20), Pharynx and larynx (1), Salivary glands (8), Unknown origin (2) Stage and histology: NA	RT (*n* = 24), CRT (*n* = 17)
Samuel, 2019 [22]	India	RCT	Exercise	148	Mean (SD) Intervention: 52.8 (9.7) Control: 52.8 (10.5)	Diagnosis (n): Larynx (28), Oropharynx (120) Stage (n): III (38), IVa (94), IVb (16) Histology: NA	All CRT
Samuel, 2013 [30]	India	RCT	Exercise	48 male 88%	Mean (SD) Intervention: 51.7 (10) Control: 52.5 (8.27)	Diagnosis: NA Stage: NA Histology: NA	All CRT
Zhao, 2016 [31]	USA	Pilot RCT	Exercise and nutrition	18 male 94%	Mean (SD) 57 (11)	Diagnosis (n): Larynx (1), Pharynx (15), Unknown origin (1) Stage (n): III (4), IV (7) Histology (n): NA	All CRT

Abbreviations: NA: not available; RT: radiotherapy; CRT: chemo/radiotherapy; RCT: randomized clinical trial.

**Table 3:** Methodological quality assessment: Randomized controlled trials on the effectiveness of exercise and/or nutrition interventions on nutritional status, physical function and quality of life in patients with head and neck cancer.

Study	Intervention Type	Criteria *											Total	Quality **
		1	2	3	4	5	6	7	8	9	10	11		
**Randomized Controlled Trials**														
Samuel, 2019 [22]	Exercise	+	+	+	+	−	−	−	+	−	+	+	7	Moderate
Samuel, 2013 [30]	Exercise	+	+	−	+	−	−	−	−	−	+	+	5	Moderate
Cereda, 2018 [23]	Nutrition	+	+	+	+	−	−	−	+	+	+	+	8	High
Jiang, 2018 [26]	Nutrition	+	+	+	+	−	−	−	+	+	+	+	8	High
Roussel, 2017 [29]	Nutrition	+	+	+	+	−	−	−	−	−	+	+	6	Moderate
Ravasco, 2005 [27]	Nutrition	?	+	+	?	−	−	−	+	+	+	+	6	Moderate
Isenring, 2003 [25]	Nutrition	−	+	?	+	−	−	?	+	−	?	+	4	Low
Hearne, 1989 [24]	Nutrition	−	?	?	?	−	−	−	−	−	+	+	2	Low
Daly, 1984 [21]	Nutrition	+	−	?	?	−	−	−	?	−	+	−	2	Low
**Pilot and Feasibility Studies**														
Sandmael, 2017 [9]	Exercise and nutrition	+	+	+	+	−	−	−	+	−	+	+	6	Moderate
Capozzi, 2016 [10]	Exercise and nutrition	+	+	+	+	−	−	+	−	+	+	−	7	Moderate
Zhao, 2016 [31]	Exercise and nutrition	+	+	+	+	+	+	+	+	+	+	+	10	High
Rogers, 2013 [28]	Exercise and nutrition	+	+	+	+	−	−	−	+	+	+	+	8	High

* The criteria addressed the following issues: 1 eligibility criteria specified; 2 randomly allocated to groups; 3 allocation concealment; 4 groups similar at baseline; 5 blinding of all subjects; 6 blinding of caregivers; 7 blinded outcome assessment; 8 measures obtained from least 85% of subjects; 9 intention-to-treat analysis; 10 between-group statistics; 11 measure of variability. + = yes, - = no and ? = unclear. Points were awarded only when a criterion was clearly satisfied. Criterion 1 is not scored. Each other criterion was given equal weight (i.e., 1 point) for a maximum sum score of 10. ** High quality: 8–10, moderate: 5–7, low: 0–4.

**Table 4:** Description of intervention, length of follow-up, adherence to intervention and completion rate, organized according to design, year of publication and intervention.

**Randomized Controlled Trials**					
**Study**	**Intervention Type**	**Description of Intervention**	**Length of Intervention**	**Intervention Adherence**	**Completion** **Rate**
Samuel, 2019 [22]	Exercise	Intervention: Brisk walking for 15–20 min and resistance training for major muscles of upper and lower limb, 2 sets and 8−15 repetitions. Exercise sessions monitored at the hospital, five days a week followed by a monitored home-based program. Control: Physical exercise recommendation, 10 min walks during the day five days a week.	Seven weeks during RT at the hospital followed by four weeks at home	NA	120/148 Lost: Intervention 16 Control 12
Samuel, 2013 [30]	Exercise	Intervention: Brisk walking for 15−20 min at perceived exertion rate between 3−5/10, five days a week. Individually tailored program for major muscle groups of upper and lower limbs 2−3 sets and 8−10 repetitions. Exercise sessions five days a week. Control: No scheduled exercise sessions but advised to remain as physically active as possible.	Intervention during RT, 6 weeks	NA	43/48 Lost: Intervention 4 Control 1
Cereda, 2018 [23]	Nutrition	Intervention: Nutritional counseling based on estimated protein−calorie requirement (1.2 g/kg of actual body weight), personal eating patterns and preferences, chewing and swallowing abilities. Addition of 1−2 bottles/day of n−3 polyunsaturated fatty acids−enriched ONS. Follow−up during RT: once a week for 6 weeks. After RT: one month and three months Control: Nutritional counseling as described above. No n−3 ONS but for ethical reasons ONS were prescribed when food intake was too low (< 60% of estimated requirements for two consecutive weeks). EN or PN was started if intake was too low for two consecutive weeks despite the use of ONS.	During RT and 3 months follow−up	NA, but protein intake (g protein/kg/day) described: End of RT: Intervention 1.0 vs. control 0.87 1 month after RT: Intervention 1.16 vs. control 0.97 3 months after RT: Intervention 1.12 vs. control 0.96	112/159 Lost: Intervention 22 Control 25
Jiang, 2018 [26]	Nutrition	Intervention: ONS 100g/day (402 kcal, 18 g protein) Control: No ONS General dietary advices in both groups every week PN with glucose if intake was severely compromised	During CRT	Consumed 52.1 g (29.4g)/day	91/100 Lost: Intervention 5 Control 4
Roussel, 2017 [29]	Nutrition	Intervention: Six individualized counselling meetings with a dietitian at home (two during RT and four at the end of RT). One meeting 2 months after end of RT. Energy and protein requirements individually evaluated and nutritional adjustments obtained with regular foods, ONS or EN if necessary. Education for self−monitoring weight, adapting intake and modifying food textures. Control: As described above but only two outpatient consultations with a dietitian during RT. Recalls if needed.	During RT, 3 months follow−up	NA but energy intake (kcal/kg/day) described: 1 month after RT: Intervention 34 vs. control 33 3 months after RT: Intervention 35 vs. control 31	87/117 Lost: Intervention 16 Control 14
Ravasco, 2005 [27]	Nutrition	Group 1 (*n* = 25): individualized counselling with regular foods Group 2 (*n* = 25): usual diet plus ONS (2 × 200 mL containing 20 g protein and 200 kcal per day) Group 3 (*n* = 25): intake ad libitum Nutritional goal for group 1 and 2 was achievement of individually calculated energy and protein requirements	Intervention during RT, 3 months follow−up	NA, but nutritional intake was primary endpoint and reported Baseline: intake similar in all groups End of RT: group 1 increase of 521 kcal/day, *p = 0.002* ONS increase of 322 kcal/day, *p = 0.05* Ad lib decrease of 400 kcal/day, *p ≤ 0.01* Between−group finding, *p = 0.005* 3 months: group 1 maintained energy intake, other groups decreased, *p = 0.001*	All completed
Isenring, 2003 [25]	Nutrition	Intervention: Individualized counselling by using a standard protocol (American Dietetic Association Medical Nutrition Therapy Head and Neck). ONS were provided when appropriate Control: Regular care, general advice by the nursing staff with samples of ONS if felt necessary.	Intervention during RT, 3 months follow−up	NA	32/36 Lost: Intervention 1 Control 3
Hearne, 1985 [24]	Nutrition	Intervention: Intensive nasogastric feeding during RT Control: Oral intake and dietary counselling Goal for intervention in both groups: 40 kcal/kg per day and 1g protein/kg per day	Intervention during RT, 1 month follow−up	Intervention: Two of 14 (14%) refused tube feeding and converted to control Control: Two of 12 (16%) converted to intervention due to weight loss Energy intake during RT (kcal/kg): Intervention 35−42 vs. control 15−34 No p−values given.	26/31 Lost: Intervention 4 Control 1
Daly, 1984 [21]	Nutrition	Intervention: EN Control: Oral intake and dietary counselling Goal in both groups: 40 kcal/kg per day and 1−1.5 g protein/kg per day. If weight gain did not occur after each week +5 kcal/kg per day. Both groups received enteral support throughout RT (approximately 8 weeks) and for several additional weeks until reaction to radiation subsided	Intervention during and up to 6 months follow−up	Intervention: Two of 22 (9%) converted to control due to non−compliance during the first week of RT Control: Two of 15 (11%) converted to tube feeding due to weight loss during the two first weeks of RT Energy intake (kcal/kg): Tube fed 39 vs. orally 30, *p < 0.00*	35/38 Lost: NA
**Pilot and Feasibility Studies**					
**Study**	**Intervention Type**	**Description of Intervention**	**Length of Intervention**	**Intervention Adherence**	**Completion** **Rate**
Sandmæl, 2017 [9]	Exercise and nutrition	Group 1: During treatment: Resistance exercises: 2 lower body− and 2 upper body, 3−4 sets, 6 to 12 repetitions, monitored by a physiotherapist at the hospital twice a week á 30 min (total 12 sessions). Recommended 150 min of moderate intensity exercise per week in addition. After the training sessions one bottle ONS. Recommended to take 1−2 ONS each day. Group 2: During treatment: Recommended to follow physical exercise guidelines for cancer patients. 2−4 weeks after end of RT: 3 weeks stay at rehabilitation centre. Resistance exercises: 3 sessions of 45 min of involving 3 upper body and 3 lower body exercises. 3−4 sets and 6 to 12 repetitions plus two voluntary sessions each week involving a combination of strength, aerobic and balance exercises with low intensity. Dietary counselling once a week in small groups and use of ONS.	Intervention during RT for group 1 and intervention after RT for group 2 Intervention initiated *during the first week* of radiotherapy lasting 6 weeks.	Adherence rates (%): Interv during RT, exercise 81 and ONS 57 After RT, exercise 94 and ONS 76	29/41 Lost: Intervention 2 Control 10
Zhao, 2016 [31]	Exercise and nutrition	Group 1: Intervention based on guidelines for patients with cancer (American College of Sports Medicine); strengthening, cardiovascular fitness and physical exercise. Exercise during the 7 weeks CRT at a clinical research center supervised by a trainer. Up to 3 sessions per week, lasting up to 1 h including warmup, cool down, and rest periods. Resistance exercises included chest press, wall push up, military press, side arm raises, biceps curl, shoulder shrugs, and calf raises. Duration and intensity were customized to the individual, goal three 8 to 12 repetition sets. Aerobic exercise was defined as walking with a pedometer and a goal to maintain step count based on the mean step count of the previous training week. Post CRT (weeks 8 to 14), integration of exercise activities into own lifestyle. Weekly telephone calls from the trainer. Before CRT counselling by a dietician, repeated in case of decrease in BMI greater than a 5% to 10%. Group 2: Standard treatment, dietary counselling and active nutritional surveillance during RT, neither encouraged nor discouraged to exercise.	Intervention for 7 weeks 7 weeks follow up	Exercise adherence rate 72%, Completed 15.2 of 21 sessions.	17/20 Lost: Intervention 1 Control 2
Rogers, 2013 [28]	Exercise and nutrition	Group 1: Nutritional counseling and 12 weeks resistance exercise. Exercise during treatment; one hour supervised sessions twice weekly at a training facility at the hospital. Six weeks of twice weekly home−based sessions supported with telephone counseling, written materials, and DVD. Up to 10 repetitions of 9 different exercises using a resistance band for major muscle groups (chest press, leg extension, lateral row, reverse curl, triceps using wall push−up/triceps kickback, heel raise, 2−arm front raise, hamstring curl, and arm curl). Intensity: light, moderate and heavy resistance bands were used. Group 2: Nutritional counseling provided by registered dietitian according to standard counseling appropriate for head and neck cancer during radiotherapy	12 weeks intervention	Exercise adherence: 6 weeks: 83% 6−12 weeks: 62% Both groups Face to face nutritional counselling (6 weeks): 96% completed Telephone counselling (6−12 weeks): 77% completed	13/15 Lost: Intervention 2 Control 0
Capozzi, 2016 [10]	Lifestyle interventions including exercise and nutrition	Group 1: 12 weeks lifestyle intervention during RT and Group 2: same intervention immediately after completion of RT. Components of intervention: physician referral and clinical support; health education; behavioral change support; individual exercise program (home exercises twice a week); group−based exercise (2 exercise sessions weekly) Exercise program: progressive resistance training with 2 sets of 8 repetitions at 8 to 10 repetitions maximum for 10 exercises targeting major muscle groups. In addition to exercise sessions participants were required to attend 6 education sessions biweekly	Immediate intervention during RT for group 1 and delayed intervention after RT for group 2 Total 24 weeks	NA	36/60 Lost: Group1: 15 Group 2: 9

Abbreviations: NA: not available; RT: radiotherapy; ONS; oral nutritional supplements; EN: enteral nutrition; PN: parenteral nutrition; CRT: chemo/radiotherapy; RCT: randomized clinical trial.

## Supplementary Materials

The following are available online at https://www.mdpi.com/2072-6643/12/11/3233/s1, Figure S1: The symmetry of body composition results presented in Funnel plot, Figure S2: The symmetry of physical function results presented in Funnel plot, Table S1: Outcomes and effects of nutritional and exercise interventions, organized according to study type, year of publication and intervention.

## Appendix A. Search Strategy

### Appendix A.1. PubMed—3 June 2019

#1. “Head and Neck Neoplasms” [Mesh] AND (“Diet Therapy” [Mesh] OR “diet therapy” [Subheading] OR “Dietary Supplements”[Mesh] OR “Exercise”[Mesh] OR “Exercise Movement Techniques”[Mesh] OR “Exercise Therapy”[Mesh])
#2. ((head[ti] OR neck[ti]) AND (cancer[ti] OR tumor[ti] OR tumour[ti] OR carcinoma*[ti])) AND (exercis*[ti] OR diet[ti] OR diets[ti] OR dietary OR nutrition*[ti] OR training[ti] OR physical activity[ti] OR rehabilitation[ti] OR life style[ti]) NOT medline[sb]
#3. #1 OR #2 > 552 hits > EndNote PubMed in label field

### Appendix A.2. Embase—1974 to 3 June 2019

1. “head and neck cancer”/dm, rt, rh, si, th [Disease Management, Radiotherapy, Rehabilitation, Side Effect, Therapy]
2. (diet therapy/or dietary intake/or exp exercise/or exp kinesiotherapy/or nutritional counseling/or nutritional support/or diet supplementation/or nutrition/)
3. 1 and 2 > 350 hits > EndNote Embase in label field

### Appendix A.3. Cochrane Library—CDSR issue 6/12, June 2019, DARE issue 2/4, April 2015, CENTRAL issue 5/12, May 2016

#1. (head or neck) and (cancer or carcinom* or tumor* or tumour*): ti,ab,kw
#2. (exercise or training or diet or diets or dietary or nutrition or rehabilitation or “life style” or “physical activity”): ti,ab,kw
#3. #1 and #2 > 379 hits (40 CDSR/7 DARE/343 CENTRAL) > EndNote CDSR/DARE/CENTRAL in label field

### Appendix A.4. CINAHL June 2019

S1 TI ((head OR neck) AND (cancer OR tumor OR tumour OR carcinoma*))
S2 AB ((head OR neck) AND (cancer OR tumor OR tumour OR carcinoma*))
S3 TI (exercis* OR diet OR diets OR nutrition* OR training OR rehabilition OR “physical activity” OR lifestyle OR “life style”)
S4 AB (exercis* OR diet OR diets OR nutrition* OR training OR rehabilition OR “physical activity” OR lifestyle OR “life style”)
S5 TI (therap* OR treatment* OR intervention* OR management* OR radiotherap* OR chemotherap* OR chemoradiotherap*)
S6 AB (therap* OR treatment* OR intervention* OR management* OR radiotherap* OR chemotherap* OR chemoradiotherap*)
S7 (s1 OR s2) AND (s3 OR s4) AND (s5 or s6) > 389 hits > EndNote CINAHL in label field
