# Supplementary material for: Exercise and Nutrition Interventions in Patients with Head and Neck Cancer during Curative Treatment: A Systematic Review and Meta-Analysis
Source: Nutrients. 2020 Oct 22;12(11):3233. doi: 10.3390/nu12113233 (PMC7690392; doi:10.3390/nu12113233)
Supplement: Supplementary file 1 [file nutrients-12-03233-s001.pdf]

Table S1. Outcomes and effects of nutritional and exercise interventions, organized according to study type, year of publication and intrevention.

Randomized controlled trials				
**Study**	**Intervention type**	**Selected outcomes**	**Measuring methods**	**Between-group finding**
Cereda, 2018	Nutrition	Body composition<br>Physical function	Total body mass<br>Handgrip dynamometer	Weight loss (kg) end of RT:<br>Intervention -1.9 vs control -3.5, $p = 0.006$<br>No statistically significant between-group difference
Jiang, 2018	Nutrition	Nutritional status<br>Body composition	PG-SGA<br>Total body mass<br>Fat-free mass by BIA	No statistically significant between-group difference<br>Weight (kg) after adjustment for baseline,<br>End of CRT: Intervention 59.11 vs control 58.14,<br>$F = 4.544$, $p \leq 0.036$<br>No statistically significant between-group difference
Roussel, 2017	Nutrition	Body composition	Total body mass<br>BMI	No statistically significant between-group differences<br>No statistically significant between-group differences
Ravasco, 2005	Nutrition	Nutritional status<br>Body composition	PG-SGA<br>Total body mass	No between-group differences given<br>No between-group differences given
Isenring, 2003	Nutrition	Body composition	Total body mass<br>Fat-free mass by BIA	Weight loss (kg) after 3 months:<br>Intervention -1.1 vs control -4.3, $p = 0.019$<br>Fat free mass loss (kg)<br>Intervention -0.3 vs control -2.2, $p = 0.029$
Hearne, 1989	Nutrition	Body composition	Total body mass	Weight loss (%) Nasopharyngeal carcinoma:<br>Intervention 3.8 vs control 3.3 *(ns)*<br>All other cancer sites: Intervention 0.2 vs control 7.3,<br>$p = 0.005$

Daly, 1984	Nutrition	Body composition	Total body mass Mid-arm muscle circumference	Weight loss (%) Nasopharyngeal carcinoma: EN 3.5 vs orally fed 5 *(ns)*. All other cancer sites: EN 0.6 vs orally 6.1, $p < 0.04$ No statistically significant between-group differences
Samuel, 2019	Exercise	Physical function	6-minute walk test	Change in walking length (meters) after 11 weeks: Intervention + 37 vs control - 73 meters, F (3,345) = 23.67, $p \leq 0.001$
Samuel, 2013	Exercise	Physical function	6-minute walk test	Change in walking length (meters) after 6 weeks: Intervention + 42 vs control - 96 meters, $p \leq 0.001$
**Pilot and feasibility studies**				
**Study**	**Intervention type**	**Selected outcomes**	**Measuring methods**	**Between-group finding**
Sandmæl, 2017	Exercise and nutrition	Body composition	Total body mass (CT)<br>Skeletal muscle mass	No statistically significant between-group differences<br>No statistically significant between-group differences
Zhao, 2016	Exercise and nutrition	Body composition<br>Physical function	BMI<br>Lean body mass (DXA)<br>Handgrip dynamometer<br>6-minute walk<br>Timed up and go	No statistically significant between-group differences<br>No statistically significant between-group differences<br>No statistically significant between-group differences<br>No statistically significant between-group differences<br>No statistically significant between-group differences
Capozzi , 2016	Lifestyle intervention including Exercise and nutrition	Nutritional status<br>Body composition<br>Physical function	PG-SGA<br>Total body mass<br>Lean body mass<br>Handgrip dynamometer<br>6-minute walk test<br>30-second sit to stand	No statistically significant between-group differences<br>No statistically significant between-group differences<br>No statistically significant between-group differences<br>No statistically significant between-group differences<br>No statistically significant between-group differences<br>No statistically significant between-group differences

Rogers, 2013	Exercise and nutrition	Body composition<br>Physical function	BMI<br>Lean body mass by BIA<br>Handgrip dynamometer<br>5 times sit to stand	Change (kg/m^2) after 6 weeks:<br>Interv -1.4 vs control -1.1, $p < 0.05$<br>No statistically significant between-group differences<br>No statistically significant between-group differences<br>No statistically significant between-group differences

Supplementary Figure.

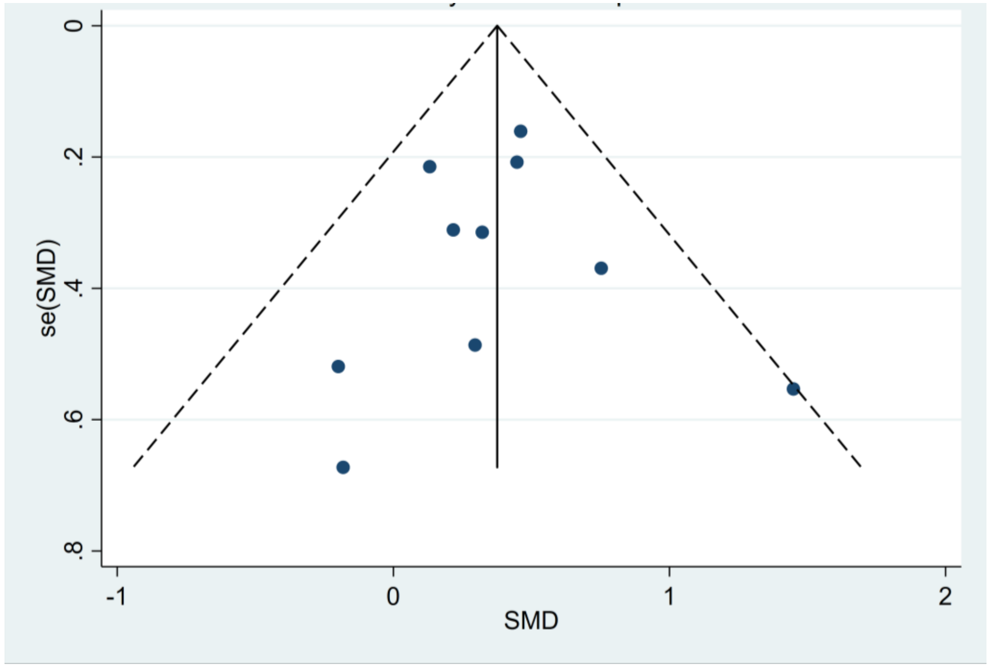

Figure S1. The symmetry of body composition results presented in Funnel plot.

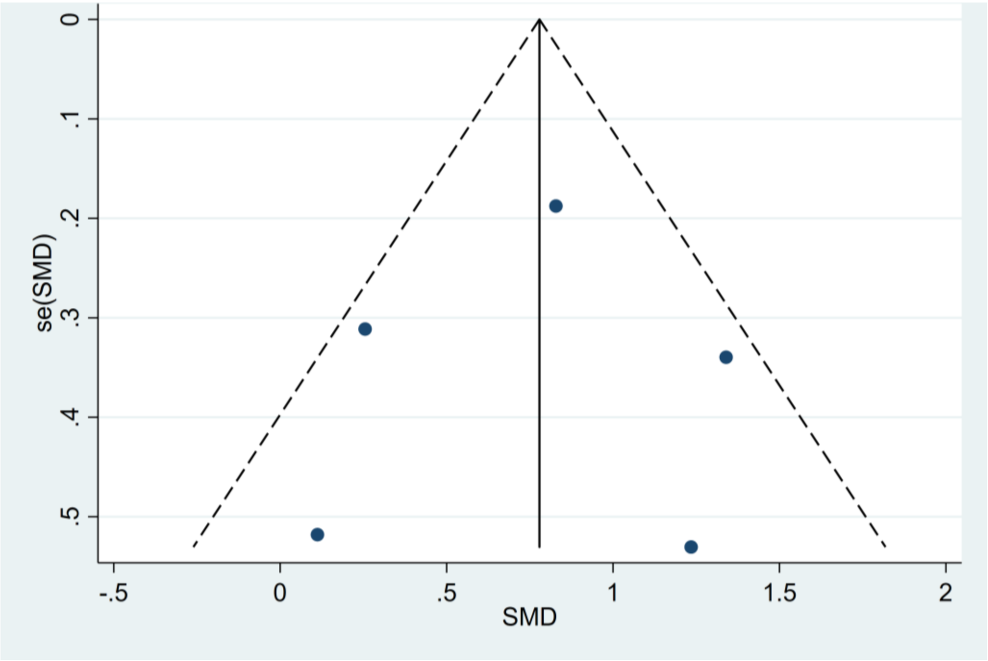

Figure S2. The symmetry of physical function results presented in Funnel plot.
